# Customer churn prediction for telecommunication industry: A Malaysian Case Study

**DOI:** 10.12688/f1000research.73597.1

**Published:** 2021-12-13

**Authors:** Nurulhuda Mustafa, Lew Sook Ling, Siti Fatimah Abdul Razak

**Affiliations:** 1 Telekom Malaysia, Faculty of Business, Ayer Keroh, Melaka, 75450, Malaysia; 2 Faculty of Information Science and Technology, Multimedia University, Ayer Keroh, Melaka, 75450, Malaysia

**Keywords:** Customer Churn, Net Promoter Score (NPS), Data Mining Techniques, Classification and Regression Trees (CART)

## Abstract

**Background**: Customer churn is a term that refers to the rate at which customers leave the business. Churn could be due to various factors, including switching to a competitor, cancelling their subscription because of poor customer service, or discontinuing all contact with a brand due to insufficient touchpoints. Long-term relationships with customers are more effective than trying to attract new customers. A rise of 5% in customer satisfaction is followed by a 95% increase in sales. By analysing past behaviour, companies can anticipate future revenue. This article will look at which variables in the Net Promoter Score (NPS) dataset influence customer churn in Malaysia's telecommunications industry.

The aim of This study was to identify the factors behind customer churn and propose a churn prediction framework currently lacking in the telecommunications industry.

**Methods**: This study applied data mining techniques to the NPS dataset from a Malaysian telecommunications company in September 2019 and September 2020, analysing 7776 records with 30 fields to determine which variables were significant for the churn prediction model. We developed a propensity for customer churn using the Logistic Regression, Linear Discriminant Analysis, K-Nearest Neighbours Classifier, Classification and Regression Trees (CART), Gaussian Naïve Bayes, and Support Vector Machine using 33 variables.

**Results**: Customer churn is elevated for customers with a low NPS. However, an immediate helpdesk can act as a neutral party to ensure that the customer needs are met and to determine an employee's ability to obtain customer satisfaction.

**Conclusions**: It can be concluded that CART has the most accurate churn prediction (98%). However, the research is prohibited from accessing personal customer information under Malaysia's data protection policy. Results are expected for other businesses to measure potential customer churn using NPS scores to gather customer feedback.

## Introduction

Customer retention and customer satisfaction are essential for a business to succeed. ^1^ Customer satisfaction is improved by repeating businesses, brand loyalty, and positive word of mouth. ^2^ Consumers prefer to stay with their current providers due to quality and price. Therefore, new anti-churn strategies must be constantly developed. ^3^ Data processing automates analytical model building. Machine learning algorithms improve the dataset iteratively to find hidden patterns. ^4^ Several studies show that machine learning can predict churn and severe problems in competitive service sectors. Predicting churning customers early on can be a valuable revenue source. ^5^ The results of the mediating effects of a customer's partial defection on the relationship between churn determinants and total defection show that some churn determinants influence customer churn, either directly or indirectly through a customer's status change, or both; thus, a customer's status change explains the relationship between churn determinants and the probability of churn. ^6^ This study hypothesised that changes in Net Promoter Score (NPS) can indicate whether churn determinants directly or indirectly influence churn.

### Telecommunication industry and service providers in Malaysia

**Figure 1.**
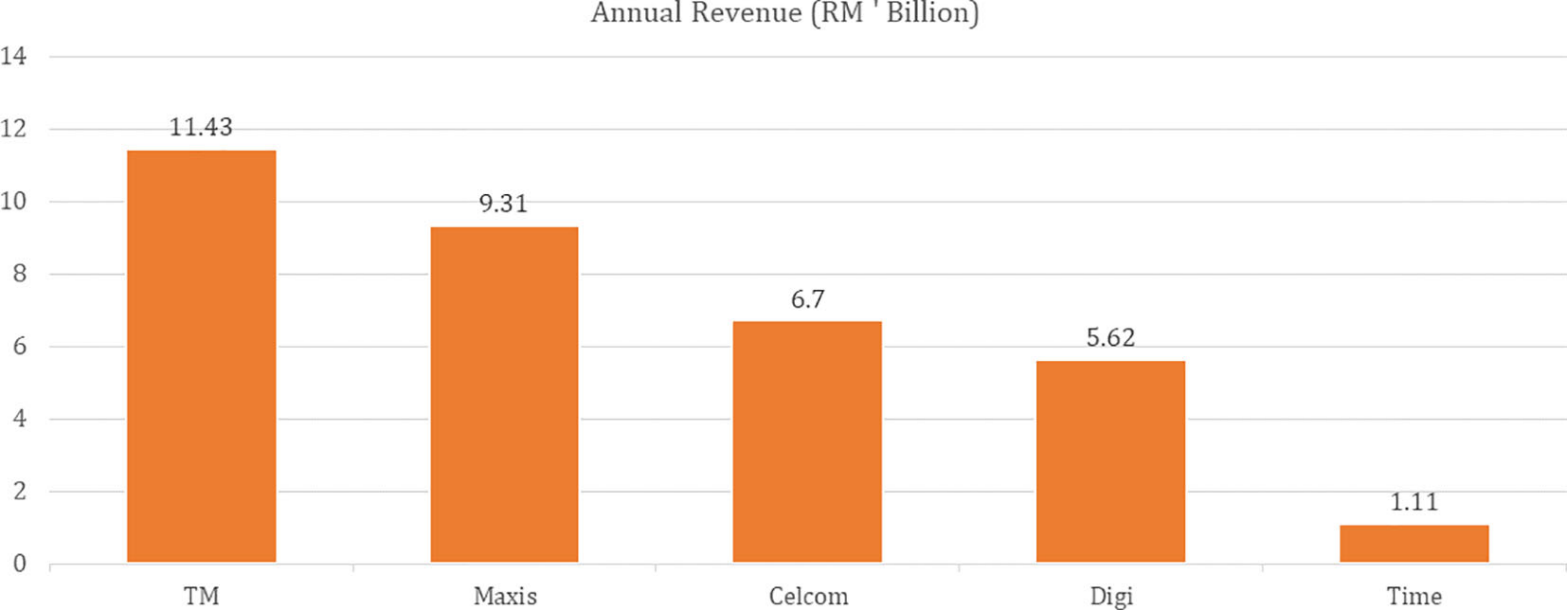
Telecommunications and Internet industry: Participants Annual Revenue as of 31 ^st^ December 2019.

Figure 1 presents the revenue generated by telecommunications in Malaysia. Telekom Malaysia (TM) has made RM11.43 million in 2019 and ranked top. ^7^

Malaysia's mobile, fixed broadband, and household penetration are expected to grow further between 2019 and 2025, providing 30 to 500 Mbps fibre internet for cities, with gigabit connections for industries. ^8^ As a result, customers expect the same or better service from providers. This statistic is also used to compare a company's performance to competitors. ^9^

### Churn rates in Malaysia

Total customer turnover is the number of customers leaving the provider. ^10^ In the telecommunications industry, market competitiveness is measured by churn rate. Telephone, internet, and mobile services are all part of telecommunications. For example, one of the Internet Service Provider (ISP) from 20 subscribers will cancel, reducing annual revenue by 5%. ^11^ Every day, the telecom industry loses 20%-40% of its customers. ^12^ Without pricing and subscription plans, 83% of Malaysians would switch telecom providers. On the other hand, 66% had no problem cancelling their existing service provider subscription. ^13^ Customer satisfaction metrics would help providers to sustain their customers.

### Net Promoter Score (NPS)

Net Promoter Score (NPS) measures customer satisfaction and loyalty using a 10-point Likert scale. ^14^ Consistency in purchases shows commitment regardless of performance and fulfilment. ^15^ Risk scores are used to predict customer churn. Churn is predicted using customer profiles and transaction patterns—predictive analytics use demographic, transactional data and NPS. ^16^ The NPS outperforms customer satisfaction. ^17^ A high NPS indicates that word of mouth can help businesses thrive. Customers are classified as promoters, detractors, or passives after receiving assistance from the helpdesk ( Table 1).

**Table 1.** Net Promoter (NPS) scale.

Scale	Score	Description
Promoters	9-10	Customers who are typically the brand's ambassadors, enhancing a brand's reputation and/publicity and referrals flow.
Passives	7-8	Customers who have positive/constructive feelings towards the brand but are not expressing a need to change.
Detractors	0-6	Customers who are unlikely to remain or encourage others to return—and even worse— may discourage others from trying to trust the business or brand.

Source: 2021 Guide & Definition (2021).

**Customer satisfaction**

Customer satisfaction is defined as customers being happy with a company's products, services, and capabilities. ^18^ Customers' satisfaction is influenced by buyer experience. ^19^ Satisfied customers lead to more sales and referrals. Proactive personal helpdesk and staff assistance require a company's ability to anticipate customer needs. Most businesses benefit from happy customers. In this study, the NPS measures customer satisfaction.

### Excellent service

An excellent service exceeds consumer expectations and satisfaction by providing high-quality services. Those are ability, attitude, appearance, attention, action, and accountability. ^20^ A customer-centric approach benefits both private and public companies. Using this mindset also helps providers win customers and save money. In addition, customers will stay loyal regardless of market choice if companies treat them well.

**Transparent subscriptions**

In 2019, accountability was the key to sustaining a profitable company. Authenticity trumps traditional priorities like price and brand recognition. ^21^ In addition, transparency promotes trust, peace of mind and openness. Happy customers are more pleasant than dissatisfied customers, As such, individual customers feedback counts.

### Churn prediction techniques/framework

Companies can classify potential customers who leave the services using advanced machine learning (ML) technology. Then, using existing data, the company can identify potential churn customers. This knowledge would allow the company to target those customers and recover them. Table 2 and Table 3 summarise the most recent churn prediction and framework studies.

**Table 2.** A summary of churn prediction studies.

Author (Year)	Techniques and method	The disadvantage of the prediction studies & proposed enhancement
Ahmad et al. (2019)	**Techniques:** Decision Tree, Random Forest, Gradient Boost Machine Tree, and XGBoost. **Method:** Churn predictive system using Hortonworks Data Platform (HDP) categorised under specific specialisation like Data Management, Data Access, Security, Operations and Governance Integration. ^22^	**Disadvantage:** Only about 5% of the dataset entries represent customer's churn. **Proposed enhancement:** The dataset entries can be solved using under-sampling or trees algorithms.
Höppner et al. (2020)	**Techniques:** Decision Tree **Method:** ProfTree learns profit-driven decision trees using an evolutionary algorithm. ProfTree outperforms traditional accuracy-driven tree-based methods in a benchmark study using real-life data from various telecommunication service providers. ^23^	**Disadvantage:** The evolutionary algorithm (EA) training times are relatively slower than other classification algorithms. **Proposed enhancement:** Combining the ProfTree algorithm with random forests further optimises property by creating profit-driven trees and aggregating them.
Yang (2019)	**Techniques:** Random Forest **Method:** A new T+2 churn customer prediction model was proposed, in which churn customers are identified in two months, and a one-month window T+1 is set aside for implementing churn management strategies. ^24^	**Disadvantage:** In the T+2 churn prediction, a precision ratio of about 50% was achieved, with a recall ratio of about 50%. **Proposed enhancement:** Proposed to use more than one algorithm technique to compare the outcome result.
Ahmed and Maheswari (2017)	**Techniques:** Firefly **Method:** Simulated Annealing modifies the actual firefly algorithm comparison to provide faster and more accurate churn predictions. ^25^	**Disadvantage:** The proposed algorithm's accuracy is comparable to that of the average firefly algorithm. **Proposed enhancement:** Incorporation of schemes or modifications to reduce False Positive rates.
Eria and Marikannan (2018)	**Techniques:** Support Vector Machines, Naïve Bayes, Decision Trees, and Neural Networks. **Method:** This study looks at 30 CCP studies from 2014-2017. Some data preparation and churn prediction issues arose. ^26^	**Disadvantage:** Telecom datasets should be handled cautiously due to their unbalanced nature, large volume, and complex structure. **Proposed enhancement:** It is necessary to investigate a gap in real-time churn prediction using big data technologies. More adaptable approaches that consider changing national or regional economic conditions are required. Customer churn could also be identified using word and voice recognition.

**Table 3.** A summary for churn prediction framework studies.

Author (Year)			
Ahn et al. (2006)	Clemes et al. (2010)	Geetha and Abitha Kumari (2012)	Kim et al. (2017)
**Propose**			
Describes a customer's status transition from active to non-user or suspended as a partial defection and from functional to absolute defective defection from active to churn. ^27^	Identifies and analyses factors influencing bank customers' switching behaviour in the Chinese retail banking industry. ^28^	Provides a brief overview of the trend of non-revenue earning customers (NRECs) that trigger revenue churn and are likely to churn soon. ^29^	Analyses the factors that are affecting IPTV service providers' behaviour regarding switching barriers, VOCs, and content consumption. ^30^
**Dataset and techniques**			
**Dataset:** 10,000 random samples from leading providers of mobile telecommunications services in South Korea **Technique:** Logistic regression	**Dataset:** 437/700 questionnaires **Technique:** Logistic regression	**Dataset:** 37,388 datasets from a leading telecom service Provider **Technique:** Linear modelling	**Dataset:** 5000 datasets from IPTV users in South Korea/ **Technique:** Logistic regression
**Variables**			
**Dependent variables** Customer churn **Independent variables** Customer Dissatisfaction Call drop rate Call failure rate Number of complaints Switching costs Loyalty points Membership card Service usage Billed amounts Unpaid balances Number of unpaid monthly bills Customer status Customer-related variables	**Dependent variables** Switching Behaviour **Independent variables** Price Reputation Service Quality Effective Advertising by The Competition Involuntary Switching Distance Switching Costs Demographic Characteristic	**Dependent variables** Susceptibility to Churn **Independent variables** The extent of Local Calls and STD Calls to Other Networks Multiple directory numbers Rate plan Tariff Admin fee Count of recharge Sum of recharge VAS Usage Total minutes of usage VAS Overall usage revenue per minute slab Total usage revenue	**Dependent variables** Customer Behaviours Customer defection **Independent variables** Switch Barrier Service bundling Remaining contract months Membership points VOC (Voice of Customer) Total VOC Membership Period Membership years Degree of Content Usage Channel views VOD views TVOD views PPV Monthly subscriptions
**Hypotheses**			
**H1** Customer Dissatisfaction à Customer churn **H2** Switching costs à Customer churn **H3** Service usage à Customer churn **H4** Customer status à Customer churn **Mediation effects** **H1'** Customer Dissatisfaction à Customer Status **H2'** Switching costs à Customer Status **H3'** Service usage à Customer Status	**H1** price à customers switching banks **H2** reputation à customers switching banks **H3** service quality à customers switching banks **H4** Effective advertising by the competition à customers switching banks **H5** Involuntary switching à customers switching banks **H6** distance à customers switching banks **H7** switching costs à customers switching banks **H8 –H15** Demographic Characteristic à customers switching banks	**H1** Proportion of local calls to other networks à susceptibility to churn **H2** Proportion of STD calls to other networks à susceptibility to churn **H3** Usage of VASs à susceptibility to churn **H4** Overall usage RPM à susceptibility to churn	**H1** Service bundling à Degree of contents usage **H2** Remaining contract months à Degree of contents usage **H3** Membership points à Degree of contents usage **H4** Total VOC à Degree of contents usage **H5** Membership years à Degree of contents usage **H6** Service bundling à Customer defection **H7** Remaining contract months à Customer defection **H8** Membership points à Customer defection **H9** Total VOC à Customer defection **H10** Membership years à Customer defection **H11** Channel views à Customer defection **H12** VOD views à Customer defection **H13** TVOD views à Customer defection **H14** PPV à Customer defection **H15** Monthly subscriptions à Customer defection
**Results**			
H1a, H1c, H2a, H3a, H4, H1c′, H2a′ and H3a′ = Supported H1b, H2b, H3b, H3c, H1a′, H1b′, H2b′, H3b′ and H3c′ = Rejected	H1 to H15 = Supported	H1 to H4 = Supported	H1, H2, H3, H4, H5, H6, H9, H14 and H15 = Supported H7, H8, H10, H11, H12 and H13 = Rejected

Machine learning can predict customer churn by identifying at-risk clients, pain points and interpreting data. Table 3 identifies dependent and independent variables in prior research on customer churn prediction. The cause is an independent variable, and the effect is a dependent variable. As such, the factors included in this study are customer churn, defection, demographics, and the voice of the customer (NPS rating).

### Traditional/statistical approaches

The traditional statistics approaches are used to solve problems involving less linear and repeatable data. They work well in environments with stable data and relationships. This is still widely used for medium- to long-term sales forecasting, where a reasonable forecast can be made with a few hundred or even fewer data points. Machine learning and statistics differ significantly. Machine learning models are created to make the most precise predictions possible. The purpose of statistical models is to make inferences about the relationships between variables. ^31^ Machine Learning is a data-driven algorithm that does not rely on rules-based programming. Statistical modelling is the use of mathematical equations to formalise relationships between variables. ^32^ This study applied machine learning and statistical approaches to analyse the potential churn and the mediation relationship between variables.

### Machine learning techniques in churn prediction

Machine Learning (ML) is a branch of artificial intelligence. ML uses existing algorithms and data sets to classify patterns. ^33^ This study adopts six widely used churn prediction techniques. All these algorithms were evaluated based on the performance accuracy when applied to the same data for a fair comparison. Table 4 describes the well-known churn prediction techniques.

**Table 4.** Churn prediction techniques.

Algorithms	Description
Logistic Regression (LR)	Logistic regression is a practical regression analysis where the dependent variable is dichotomous; (binary). Logistic regression is used to characterise data and explain the relationship between one dependent binary variable and one or more nominal, ordinal, interval, or ratio-level independent variables. The odds ratio of multiple explanatory variables is calculated by logistic regression. Except that the response variable is binomial, the method is like multiple linear regressions. The result is the impact of each variable on the odds ratio of the event of interest. ^34^
Linear Discriminant Analysis (LDA)	Linear Discriminant Analysis, or LDA, is a technique used to minimise dimensionality. It is used as a pre-processing stage in applications for ML and pattern classification. The LDA aims to project the functions into a lower-dimensional space in a higher-dimensional space to avoid the curse of dimensionality and minimise energy and dimensional costs. ^35^
K-Nearest Neighbours Classifier (KNN)	The Nearest Neighbour Classifier is a classification accomplished by defining the nearest neighbours as an example of a query and using those neighbours to evaluate the query's class. This classification approach is of particular interest since common run-time efficiency concerns are not the available computing resources these days. ^36^
Classification and Regression Trees (CART)	Classification of and Regression Trees is a classification scheme that uses historical data to construct so-called decision trees. Decision trees can then be used in the form of new outcomes. First, the CART algorithm will search for all possible variables and all possible values to find the best partition—a query that divides the data into two parts with the highest homogeneity. Then, the process is repeated for each of the resulting data fragments. ^37^
Gaussian Naive Bayes (NB)	Gaussian Naive Bayes is a particular case of probabilistic networks that allows the treatment of continuous variables. It is a generalisation of Naive Bayes Networks. The Naïve Bayes Classifiers are based on the Theorem of Bayes. One of the assumptions taken is the apparent presumption of freedom between functions. Furthermore, these classifiers assume that a particular function's value is unaffected by the value of any other feature. Therefore, naive Bayed Classifiers require a small amount of training data. ^38^
Support Vector Machine (SVM)	Support vector machines (SVMs) are supervised learning methods known as regression, used for classification. Support vector machine (SVM) uses machine learning theory to classifier and regression prediction to maximise predictive accuracy while preventing overfitting the training. In general, SVMs may be thought of as systems that utilise functions in a high-dimensional feature space and are taught using an optimisation theory-based learning method that promotes statistical learning. ^39^

## Research model

### Customer churn determinants

**Figure 2.**
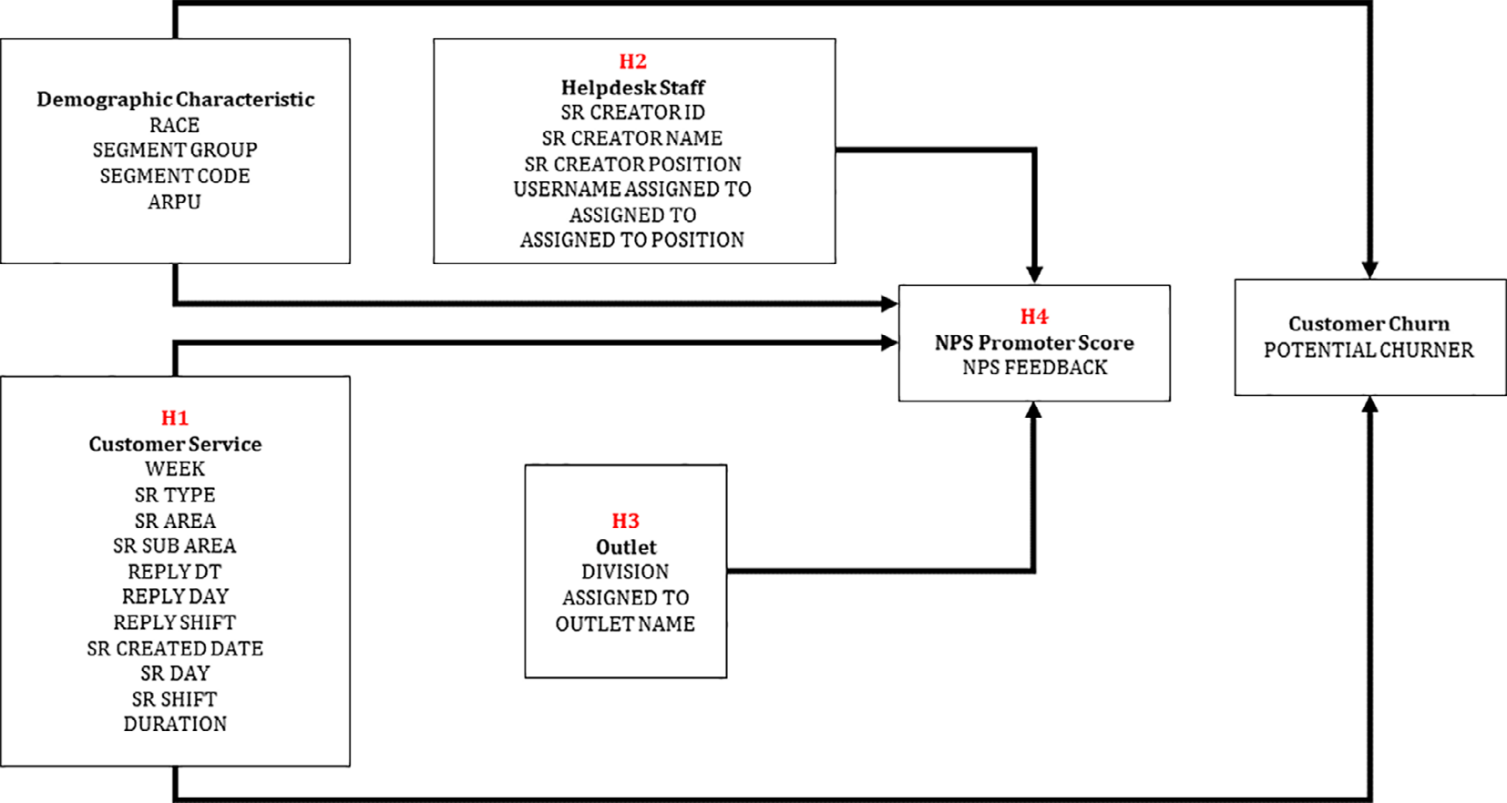
A conceptual model for the prediction of potential churner.

The following paragraphs explain the determinants of customer churn considered in this study. Figure 2 depicts four primary structures that may influence potential customer churn and the indirect effects of NPS feedback.

H1: Service Request

Companies recognise that poor customer service jeopardises customer relationships and revenue in the highly competitive telecommunications industry. Therefore, the degree to which telecommunications companies disconnect services indicates customer satisfaction and is directly proportional to customer turnover ^40^ ( Table 5).

**Table 5.** H1 Hypothesis.

H1a	WEEK is positively associated with the customer churn probability
H1b	SR TYPE is positively associated with the customer churn probability
H1c	SR AREA is positively associated with the customer churn probability
H1d	REPLY DT are positively associated with the customer churn probability
H1e	REPLY DAY are positively associated with the customer churn probability
H1f	REPLY SHIFT is positively associated with the customer churn probability
H1g	SR CREATED DATE is positively associated with the customer churn probability
H1h	SR DAY are positively associated with the customer churn probability
H1i	SR SHIFT is positively associated with the customer churn probability
H1j	DURATION is positively associated with the customer churn probability

As competition grows and consumers place a higher value on service quality, service providers may find it increasingly difficult to succeed unless they pay greater attention to consumer reviews and concerns. ^41^

H2: Helpdesk Staff

Several customers stop doing business with a company when they feel unappreciated, unable to get the information they want to speak to them or an unreasonably rude and unhelpful employee ^42^ ( Table 6).

**Table 6.** H2 Hypothesis.

H2a	SR CREATOR ID is positively associated with the customer churn probability
H2b	SR CREATOR NAME is positively associated with the customer churn probability
H2c	SR CREATOR POSITION is positively associated with the customer churn probability
H2d	USERNAME ASSIGNED TO are positively associated with the customer churn probability
H2e	ASSIGNED TO are positively associated with the customer churn probability
H2f	ASSIGNED TO POSITION are positively associated with the customer churn probability

Poor customer service, such as rude employees, delays in service, or incorrect details, can cause customer frustration and increase the churn rate. ^43^

H3: Outlet

Professionalism, friendliness, knowledge, communication, and sales skills are a few examples. Additionally, providers can reduce customer churn by adjusting service prices, policies, and branching ^44^ ( Table 7).

**Table 7.** H3 Hypothesis.

H3a	DIVISION ASSIGNED TO are positively associated with the customer churn probability
H3b	OUTLET NAME is positively associated with the customer churn probability

An important management assumption is that employee attitudes and reactions to organisational changes are related to department performance. ^45^

H4: NPS score feedback

Collecting NPS surveys is a great way to get customer feedback and send them to the right team. For example, promoters should send a customer's name to the team for testimonials and case studies or sign up for a customer loyalty programme. ^46^ The survey divided over a thousand NPS feedback types into three categories: distractor, passive, and promoter ( Table 8).

**Table 8.** H4 Hypothesis.

H4	A lower NPS feedback rating is considered more potential churner than a customer with a higher NPS feedback rating

### Mediation effects of NPS feedback

A mediating variable links the independent and dependent variables. Its existence explains why the other two variables have a mediator relationship. ^47^ Some churn predictors may impact customer churn directly, indirectly, or both. This study defines partial defection as an NPS feedback rating from promoter to passive and total defection as passive to the distractor. Thus, it acts as a link between churn predictors and customer loss. Partial defection's mediation effects on churn determinants and total defection are investigated. Hence, an NPS feedback status is hypothesised to mediate the relationship ( Table 9).

**Table 9.** Mediation effects on NPS feedback.

H1a'	A NPS Feedback rating mediates the effect of request week on customer churn.
H1b'	A NPS Feedback rating mediates the effect of service request type on customer churn.
H1c'	A NPS Feedback rating mediates the effect of the service request area on customer churn
H1d'	A NPS Feedback rating mediates the effect of respond date on customer churn
H1e'	A NPS Feedback rating mediates the effect of respond day on customer churn
H1f'	A NPS Feedback rating mediates the effect of respond day shift on customer churn
H1g'	A NPS Feedback rating mediates the effect of service request date on customer churn
H1h'	A NPS Feedback rating mediates the effect of service request day on customer churn
H1i'	A NPS Feedback rating mediates the effect of service request day shift on customer churn
H1j'	A NPS Feedback rating mediates the effect of service duration on customer churn.
H2a'	A NPS Feedback rating mediates the effect of service request created staff ID on customer churn
H2b'	A NPS Feedback rating mediates the effect of service request created staff name on customer churn
H2c'	A NPS Feedback rating mediates the effect of service request created staff position on customer churn
H2d'	A NPS Feedback rating mediates the effect of assigned staff ID on customer churn
H2e'	A NPS Feedback rating mediates the effect of assigned staff name on customer churn
H2f'	A NPS Feedback rating mediates the effect of assigned staff position on customer churn
H3a'	A NPS Feedback rating mediates the effect of assigned division on customer churn
H3b'	A NPS Feedback rating mediates the effect of the assigned outlet on customer churn

## Methods

This empirical study used 7776 random samples from a database of one of Malaysia's leading telecommunications service providers, spanning from two datasets ((MTD) September 2019 and (MTD) September 2020)) ( Table 10). The primary key results from the discovery process can be seen in Table 11.

**Table 10.** Dataset details.

No	Data	Data type	Original/adding data	Details
1	SR NUMBER	object	Original data	Service request tracking number
2	WEEK	int64	Original data	Number of weeks (year)
3	CUSTOMER NAME	object	Original data	Customer/Business Name
4	RACE	object	Add data	Customer Race
5	REPLY DT	object	Original data	Respond Date
6	REPLY DAY	object	Add data	Respond Day
7	REPLY SHIFT	object	Add data	Respond Day Shift
8	S_NPS_FEEDBACK	int64	Original data	NPS Feedback Rating (0-10)
9	S_NPS_FEEDBACK_TYPE_FK	object	Original data	NPS Feedback Type (Promoter, Passive, Distractor)
10	NES RESPONSE	object	Original data	NPS comment
11	SEGMENT GROUP	object	Original data	Customer segmentation group (consumer, SME's, government, and enterprise)
12	SEGMENT CODE	object	Original data	Customer segmentation code
13	ARPU	float64	Add data	The average revenue per user (customer)
14	SR CREATED DATE	object	Original data	Service Request Date
15	SR DAY	object	Add data	Service Request Day
16	SR SHIFT	object	Add data	Service Request Day shift
17	DURATION	object	Add data	Respond Time duration
18	SR TYPE	object	Original data	Service Request Type
19	SR AREA	object	Original data	Service Request Area
20	SR SUB AREA	object	Original data	Service Request Sub Area
21	SR CREATOR ID	object	Original data	Helpdesk Staff Creator ID
22	SR CREATOR NAME	object	Original data	Helpdesk Staff Creator Name
23	CREATOR POSITION	object	Original data	Helpdesk Staff Creator Position
24	USERNAME ASSIGNED TO	object	Original data	Officer in Charge Username
25	ASSIGNED TO	object	Original data	Officer in Charge Name
26	ASSIGNED TO POSITION	object	Original data	Officer in Charge Position
27	DIVISION ASSIGNED TO	object	Original data	Assigned Division
28	BUILDING ID	object	Original data	Assigned Outlet ID
29	OUTLET NAME	object	Original data	Assigned Outlet Name
30	ZONE	object	Original data	Assigned Outlet Zone
31	STATE	object	Original data	Assigned Outlet State
32	SOURCE	object	Original data	Service Request System
33	POTENTIAL CHURNER	object	Add data	Potential churner or not

**Table 11.** Key findings from the discovery process.

MTD Sept 2019	MTD Sept 2020
**Total Dataset:** 4554 Potential Churner: No: 4307 (94.58%) Yes: 247 (5.42%)	**Total Dataset:** 3222 Potential Churner: No: 3101 (96.24%) Yes: 121 (3.76%)
**Customer segmentation** Consumer: 4036, potential churner 219 = 5.4% Enterprise: 18, potential churner 1 = 5.6% Gorvernment: 24, potential churner 0 = 0% SME: 476, potential churner 27 = 5.7% From the total 4554 customers, Consumer segmentation is the highest segmentation interact with helpdesk staff and contribute to NPS feedback rating = 88.6%	**Customer segmentation** Consumer: 2762, potential churner 102 = 3.7% Enterprise: 19, potential churner 1= 5.6% Gorvernment: 20, potential churner 2 = 10% SME: 419, potential churner 16 = 3.8% From the total 3222 customers, Consumer segmentation is the highest segmentation interact with helpdesk staff and contribute to NPS feedback rating = 85.7%
**NPS feedback rating** Promoter: 3665, given a rating between 9-10 Passive: 577, given a rating between 7-8 Detractor: 312, given a rating between 0-6, potential churner 247 = 79.2% equivalence to given rating between 0-5 9-10 (promoter) is the highest rating given by customer = 80.5% from overall 4554 customers	**NPS feedback rating** Promoter: 2963, given a rating between 9-10 Passive: 125, given a rating between 7-8 Detractor: 134, given a rating between 0-6, potential churner 121 = 90.3% equivalence to given rating between 0-5 9-10 (promoter) is the highest rating given by customer = 92.1% from overall 3222 customers
**Numerical value** Week: the majority of 1271 customers communicates with the helpdesk staff in week 39, 3 ^rd^ week of Sept 2019 NPS feedback: the majority of 2436 customers were given a rating of 10. Average revenue per user (ARPU): 1275, R40 customers with APPU RM116.57 (Consumer) is the majority segmentation group interact with helpdesk staff.	**Numerical value** Week: the majority of 794 customers communicates with the helpdesk staff in week 39, 3 ^rd^ week of Sept 2019 NPS feedback: the majority of 2494 customers were given a rating of 10. Average revenue per user (ARPU): 1221, R40 customers with APPU RM116.40 (Consumer) is the majority segmentation group interact with helpdesk staff.

### Variable selection and transformation

The dependent variables for potential churner are binary, with 1 representing “yes” and 0 representing “no”. In addition, a multinomial variable for each account indicates promoter, passive, and distractor NPS feedback. Thus, a positive correlation coefficient implies a direct relationship between the two variables. Conversely, inverse correlation occurs when one variable rises while the other falls. ^48^ Finally, after data pre-processing, eight irrelevant variables were dropped, and 25 variables were selected and converted numerically to avoid unstable coefficient estimates and difficult model interpretation ( Table 12) ( *Underlying data*). ^49^

**Table 12.** Variable selection and transformation.

No	Data	Data type
1	WEEK	int64
2	RACE	int64
3	REPLY DT	int64
4	REPLY DAY	int64
5	REPLY SHIFT	int64
6	S_NPS_FEEDBACK	int64
7	S_NPS_FEEDBACK_TYPE_FK	int64
8	SEGMENT GROUP	int64
9	SEGMENT CODE	int64
10	ARPU	float64
11	SR CREATED DATE	int64
12	SR DAY	int64
13	SR SHIFT	int64
14	DURATION	int64
15	SR TYPE	int64
16	SR AREA	int64
17	SR CREATOR ID	int64
18	SR CREATOR NAME	int64
19	SR CREATOR POSITION	int64
20	USERNAME ASSIGNED TO	int64
21	ASSIGNED TO	int64
22	ASSIGNED TO POSITION	int64
23	DIVISION ASSIGNED TO	int64
24	OUTLET NAME	int64
25	POTENTIAL CHURNER	int64

**Figure 3.**
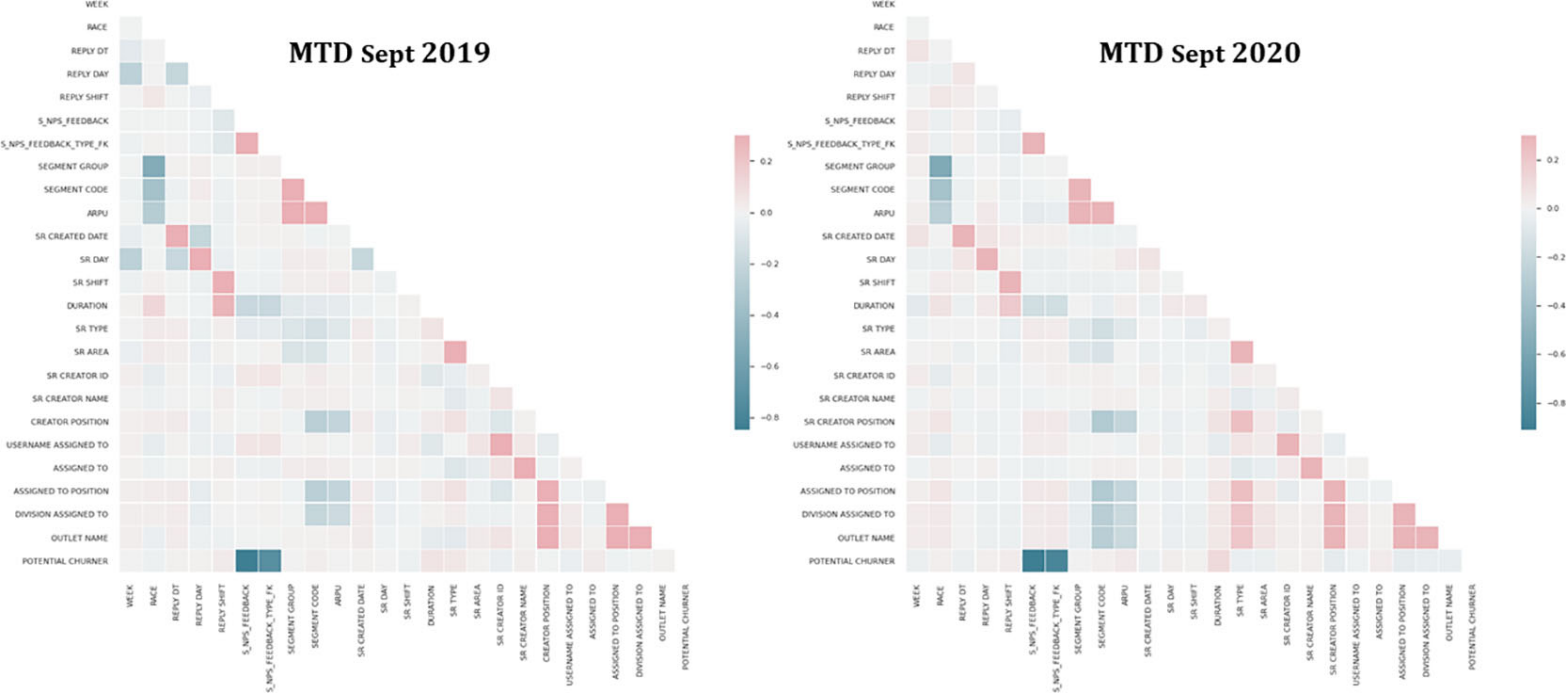
Correlation coefficient results for MTD Sept 2019 and MTD Sept 2020.

The study found high correlations between variables. Figure 3 shows the most positive correlation between helpdesk staff and assigned officer in charge (r = 0.98 & 0.96) and the most negative correlation between NPS feedback and potential churner (r = −0.85 & −0.91). The potential churner is found to be negatively related to NPS Feedback. Customers with lower NPS ratings are more likely to churn than those with higher ratings.

## Results

### Machine learning algorithm

This study tested a harness to use 10-fold cross-validation, builds multiple models to predict measurements, and selects the best model. As a result, CART has the highest estimated accuracy score of 0.98 or 98% ( Table 13).

**Table 13.** Machine learning algorithms comparison results.

Algorithms Name	MTD Sept 2019			MTD Sept 2020		
	Mean	Std	Accuracy score	Mean	Std	Accuracy score
Logistic Regression (LR)	0.42	0.01	41%	0.44	0.01	45%
Linear Discriminant Analysis (LDA)	0.41	0.02	42%	0.47	0.02	45%
K-Nearest Neighbours Classifier (KNN)	0.98	0.01	98%	0.98	0.01	97%
Classification and Regression Trees (CART)	0.98	0.01	98%	0.98	0.01	98%
Gaussian Naive Bayes (NB)	0.41	0.01	41%	0.44	0.02	44%
Support Vector Machine (SVM)	0.98	0.01	98%	0.96	0.01	98%

**Figure 4.**
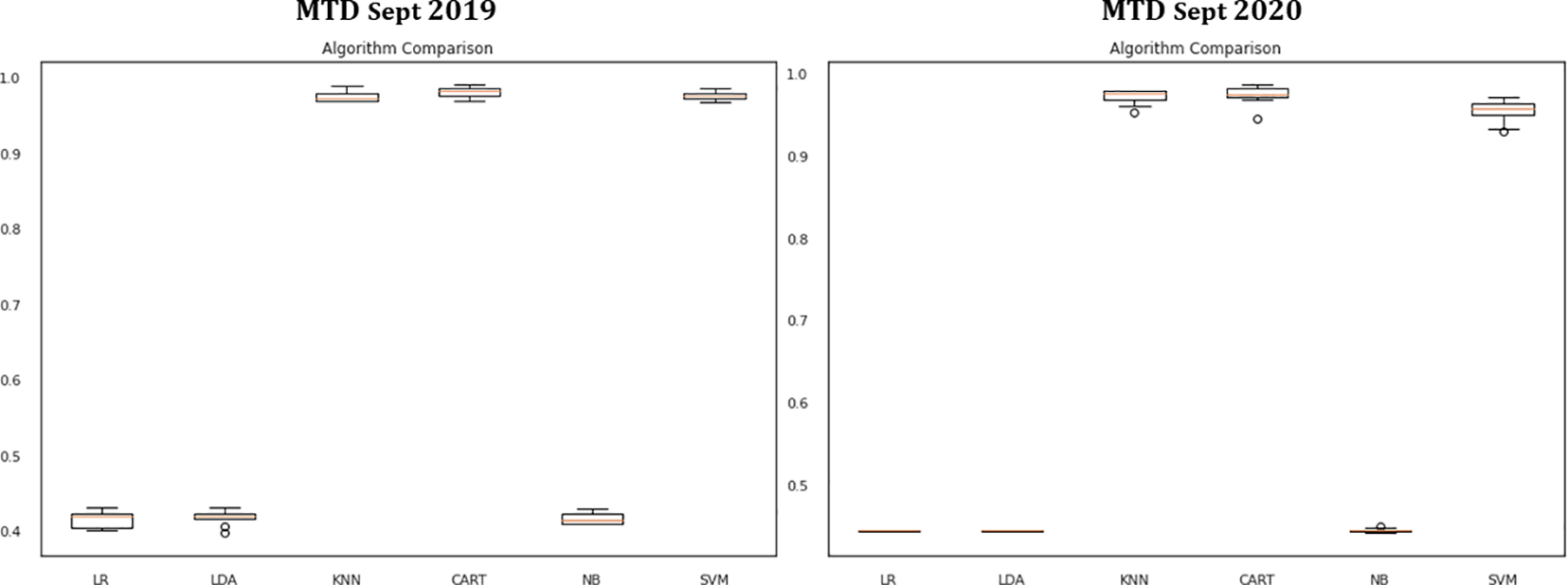
Comparing machine learning algorithms for MTD Sept 2019 and MTD Sept 2020.

In Figure 4, the box and whisker plots at the top of the range, with CART, SVM, and KNN evaluations achieving 100% accuracy and NB, LDA, and LR evaluations falling into the low 41% accuracy range.

### Mediation analysis results

Table 14 shows the findings of the mediation effects, with statistical significance presented as a p-value less than 0.05. According to the results of this study, the NPS feedback rating appears to be a partial mediator between some churn determinants and customer churn. NPS Feedback is found to be a significant mediator of several churn determinants. The NPS feedback rating change partially mediates the following variables' effects on the probability of customer churn, Duration, Reply Shift, Service Request Type, Helpdesk Staff ID, and Assigned Officer to handle the task have a significant relationship with potential churn customer.

**Table 14.** Mediation analysis results.

X: Independent variable				
M: NPS feedback				
Y: Potential churner				
X	X and M (p-value)	X and Y (p-value)	X, M and Y (p-value) sobel test	Significant relationship X and Y via M
WEEK	0.5365	0.7852	0.5365	No
SR TYPE *	0.0001	0.0024	0.0001	Yes
SR AREA	0.6196	0.7088	0.6195	No
REPLY DT	0.5634	0.8215	0.5634	No
REPLY DAY	0.4331	0.4755	0.4331	No
REPLY SHIFT *	0.0001	0.0138	0.0001	Yes
SR CREATED DATE	0.7494	0.8044	0.7494	No
SR DAY	0.7311	0.7859	0.7311	No
SR SHIFT	0.4008	0.2146	0.4008	No
DURATION *	0.0001	0.0001	0.0001	Yes
SR CREATOR ID *	0.0009	0.0653	0.0009	Yes
SR CREATOR NAME	0.5596	0.1048	0.5596	No
SR CREATOR POSITION	0.7382	0.7535	0.7382	No
USERNAME ASSIGNED TO *	0.0002	0.0292	0.0002	Yes
ASSIGNED TO	0.1727	0.0206	0.1727	No
ASSIGNED TO POSITION	0.9376	0.4814	0.9376	No
DIVISION ASSIGNED TO	0.9622	0.6390	0.9622	No
OUTLET NAME	0.6197	0.5280	0.6197	No

* p-value (<.05).

### Hypothesis test results: Customer churn determinants

H1b reveals that SR TYPE (Service Request Type) has a significant impact on the probability of churn ( Table 15). This finding is supported by Solution Partner (2019) that customers' requests should be centralised to avoid multiple ticket opening sources. One-stop-centre to answer requests, provide helpful information, and engage with customers until the problem is solved. ^50^

**Table 15.** Summary of hypothesis results.

Hypothesis	Description	Decision
H1a	WEEK is positively associated with the customer churn probability	Rejected
H1b	SR TYPE is positively associated with the customer churn probability	Supported
H1c	SR AREA is positively associated with the customer churn probability	Rejected
H1d	REPLY DT are positively associated with the customer churn probability	Rejected
H1e	REPLY DAY are positively associated with the customer churn probability	Rejected
H1f	REPLY SHIFT is positively associated with the customer churn probability	Supported
H1g	SR CREATED DATE is positively associated with the customer churn probability	Rejected
H1h	SR DAY are positively associated with the customer churn probability	Rejected
H1i	SR SHIFT is positively associated with the customer churn probability	Rejected
H1j	DURATION is positively associated with the customer churn probability	Supported
H2a	SR CREATOR ID is positively associated with the customer churn probability	Supported
H2b	SR CREATOR NAME is positively associated with the customer churn probability	Rejected
H2c	SR CREATOR POSITION is positively associated with the customer churn probability	Rejected
H2d	USERNAME ASSIGNED TO are positively associated with the customer churn probability	Supported
H2e	ASSIGNED TO are positively associated with the customer churn probability	Rejected
H2f	ASSIGNED TO POSITION are positively associated with the customer churn probability	Rejected
H3a	DIVISION ASSIGNED TO are positively associated with the customer churn probability	Rejected
H3b	OUTLET NAME is positively associated with the customer churn probability	Rejected
H4	A lower NPS feedback rating is considered more potential churner than a customer with a higher NPS feedback rating	Supported

H1f reveals that REPLY SHIFT (Respond Day Shift) significantly impacts the probability of churn. It is consistent with past research; the speed of customer service responses is also important. Thus, the solution can influence employee and customer engagement when seeking solutions. ^51^

H1j reveals that DURATION (Respond Time Duration) has a significant impact on the probability of churn. This result supports Scout (2020) that time is an important factor in determining customer service quality. According to Forrester Research, 77% of customers believe that respecting their time is the most important online customer service. ^52^

H2d reveals that USERNAME ASSIGNED (Officer in Charge Username) significantly impacts the probability of churn. However, H2f does not. As a result, help desk staff must assign problems to experts based on case-by-case categories. Adebiyi *et al.* (2016) found that failing to respond to customer complaints or provide solutions may result in poor service delivery and contract termination. ^53^

## Discussion

This study found that customer satisfaction with helpdesk service affects NPS scores. Each rating meets customers' expectations. Understanding the provider's potential churner will also reveal how the company operates, whether it provides a high-quality product with excellent customer service or needs to improve significantly to compete.

It is important to acknowledge customers' contributions to the product or service's value. Responding to complaints is a good start, but if the provider wants to stay in business, they will need to do more. Providers will be delighted if they meet all customers' needs while providing the highest quality services. ^54^

From 7776 records, 5% of customers with NPS ratings below 6 are potential churners. The Customer Relationship Management (CRM) team will find potential churners with the initiative and techniques to retain customers using the same framework. Pope (2020) research the customers' lifetime value entirely depends on how hard the businesses work to maintain them. Providing a personalised customer experience will keep them coming back. It will also turn ardent supporters into online, social media, and in-person brand advocates. But building the brand momentum through happy customers takes time and effort. A good product is not enough. When consumers make purchases, they anticipate an experience. Numerous businesses use retention marketing to ensure brand consistency. The third and final phase, retention marketing, is the most critical. Providers must focus on customer relationships. ^55^

## Conclusions

This research used data from September 2019 and September 2020 to predict churn potential among Malaysian telecommunications customers. According to the results, the immediate helpdesk response can ensure that customers' needs are met and act as mediators in determining an employee's ability to satisfy customers. This study evaluated six machine learning algorithms, with CART having the most accurate performance (98%). The NPS feedback rating partially mediates customer churn. The proposed framework will help providers accurately predict potential churn customers and help CRM teams offer targeted churning customers win-back programmes. For better findings and analysis, more research should be done on NPS rating and provided customer feedback.

## Data availability

### Underlying data

Zenodo: Customer Churn Prediction for telecommunication Industry: A Malaysian Case Study.

DOI: https://doi.org/10.5281/zenodo.5758742. ^49^

This project contains the following underlying data:

• Dataset MTD Sept 2019.csv (The file contains Net Promoter Score (NPS) in Month to Date (MTD) September 2019 of a telecommunication company. 25 variables were used to determine potential churn customer).
• Dataset MTD Sept 2020.csv (The file contains Net Promoter Score (NPS) in Month to Date (MTD) September 2020 of a telecommunication company. 25 variables were used to determine potential churn customer).

Data are available under the terms of the Creative Commons Attribution 4.0 International license (CC-BY 4.0).

## Author contributions

Nurulhuda, M., Lew, S. L., & Siti, F. A. R. comprehended the idea and contributed to the research article. All authors contributed to the writing, editing, and consent of the final manuscript.
