# Supplementary material for: Customer churn prediction for telecommunication industry: A Malaysian Case Study
Source: F1000Res. 2021 Dec 13;10:1274. [Version 1] doi: 10.12688/f1000research.73597.1 (PMC9051585; doi:10.12688/f1000research.73597.1)
Supplement: Peer review history

# Peer review: Reviewer response for version 1

**DOI:** 10.5256/f1000research.77258.r129495

**Published:** 2022-04-28

**Authors:** D. I. George Amalarethinam

**Affiliations:** 1 Department of Computer Science, Jamal Mohamed College, Tiruchirappalli, Tamil Nadu, India

The salient features of gait are analysed to predict churn potential using the data from one of the Malaysia's standard telecommunications service providers with the specific period from September 2019 to September 2020.

- Additional explanation to be provided towards data called for in tables.
- Though the paper is technically sound, the limitations or negative aspects of the proposed methodology needs to be included.
- The recent year references may be provided towards strengthening of the paper.

Is the work clearly and accurately presented and does it cite the current literature?

Yes

If applicable, is the statistical analysis and its interpretation appropriate?

Not applicable

Are all the source data underlying the results available to ensure full reproducibility?

Yes

Is the study design appropriate and is the work technically sound?

Yes

Are the conclusions drawn adequately supported by the results?

Partly

Are sufficient details of methods and analysis provided to allow replication by others?

Partly

Reviewer Expertise:

Cloud Scheduling & Security, Data Mining, Network Security

I confirm that I have read this submission and believe that I have an appropriate level of expertise to confirm that it is of an acceptable scientific standard.

# Peer review: Reviewer response for version 1

**DOI:** 10.5256/f1000research.77258.r109266

**Published:** 2022-01-04

**Authors:** Catur Supriyanto

**Affiliations:** 1 Faculty of Computer Science, Universitas Dian Nuswantoro, Semarang, Indonesia

The research paper title on “Customer churn prediction for telecommunication industry: A Malaysian Case Study” is well organized and the originality of the paper is good. The main objective of the paper is to compare some machine learning (ML) algorithms for customer churn prediction in telecommunication industry.

 In experiment the authors analyzed that the data were collected from one of the Malaysia's leading telecommunications service providers. The authors hypothesized that changes in Net Promoter Score (NPS) can indicate whether churn determinants directly or indirectly influence churn. Hence, they decided to execute many algorithms such as Logistic Regression, K-Nearest Neighbor (KNN), Support Vector Machine (SVM), Linear Discriminant Analysis (LDA), Classification and Regression Trees (CART), Gaussian Naive Bayes (NB) algorithms.

 The authors concluded that the CART outperforms with an accuracy of 98% than other ML algorithms. The structure of the paper and result findings are good. Finally, the outcome of the paper is good and suggested to other businesses to measure potential customer churn using NPS scores to gather customer feedback in near future.

 Hence, I have recommended this paper to be indexed.

Is the work clearly and accurately presented and does it cite the current literature?

Yes

If applicable, is the statistical analysis and its interpretation appropriate?

Yes

Are all the source data underlying the results available to ensure full reproducibility?

Yes

Is the study design appropriate and is the work technically sound?

Yes

Are the conclusions drawn adequately supported by the results?

Yes

Are sufficient details of methods and analysis provided to allow replication by others?

Yes

Reviewer Expertise:

Machine learning

I confirm that I have read this submission and believe that I have an appropriate level of expertise to confirm that it is of an acceptable scientific standard.
